# A Novel Nomogram including AJCC Stages Could Better Predict Survival for NSCLC Patients Who Underwent Surgery: A Large Population-Based Study

**DOI:** 10.1155/2020/7863984

**Published:** 2020-05-20

**Authors:** Xiaoling Shang, Haining Yu, Jiamao Lin, Zhenxiang Li, Chenglong Zhao, Jian Sun, Haiyong Wang

**Affiliations:** ^1^Department of Clinical Laboratory, Shandong University, Jinan 250012, China; ^2^Department of Clinical Laboratory, Shandong Cancer Hospital and Institute, Shandong First Medical University and Shandong Academy of Medical Sciences, Jinan 250117, China; ^3^Personnel Division, Shandong Cancer Hospital and Institute, Shandong First Medical University and Shandong Academy of Medical Sciences, Jinan 250117, China; ^4^Department of Internal Medicine-Oncology, Shandong Cancer Hospital and Institute, Shandong First Medical University and Shandong Academy of Medical Sciences, Jinan 250117, China; ^5^Department of Radiation Oncology, Shandong Cancer Hospital and Institute, Shandong First Medical University and Shandong Academy of Medical Sciences, Jinan 250117, China; ^6^Department of Pathology, Shandong Cancer Hospital and Institute, Shandong First Medical University and Shandong Academy of Medical Sciences, Jinan 250117, China; ^7^Department of Thoracic Surgery, Shandong Cancer Hospital and Institute, Shandong First Medical University and Shandong Academy of Medical Sciences, Jinan 250117, China

## Abstract

**Objective:**

In this study, we aimed to establish a novel nomogram model which was better than the current American Joint Committee on Cancer (AJCC) stage to predict survival for non-small-cell lung cancer (NSCLC) patients who underwent surgery. *Patients and Methods*. 19617 patients with initially diagnosed NSCLC were screened from Surveillance Epidemiology and End Results (SEER) database between 2010 and 2015. These patients were randomly divided into two groups including the training cohort and the validation cohort. The Cox proportional hazard model was used to analyze the influence of different variables on overall survival (OS). Then, using R software version 3.4.3, we constructed a nomogram and a risk classification system combined with some clinical parameters. We visualized the regression equation by nomogram after obtaining the regression coefficient in multivariate analysis. The concordance index (C-index) and calibration curve were used to perform the validation of nomogram. Receiver operating characteristic (ROC) curves were used to evaluate the clinical utility of the nomogram.

**Results:**

Univariate and multivariate analyses demonstrated that seven factors including age, sex, stage, histology, surgery, and positive lymph nodes (all, *P* < 0.001) were independent predictors of OS. Among them, stage (C-index = 0.615), positive lymph nodes (C-index = 0.574), histology (C-index = 0.566), age (C-index = 0.563), and sex (C-index = 0.562) had a relatively strong ability to predict the OS. Based on these factors, we established and validated the predictive model by nomogram. The calibration curves showed good consistency between the actual OS and predicted OS. And the decision curves showed great clinical usefulness of the nomogram. Then, we built a risk classification system and divided NSCLC patients into two groups including high-risk group and low-risk group. The Kaplan–Meier curves revealed that OS in the two groups was accurately differentiated in the training cohort (*P* < 0.001). And then, we validated this result in the validation cohort which also showed that patients in the high-risk group had worse survival than those in the low-risk group.

**Conclusion:**

The results proved that the nomogram model had better performance to predict survival for NSCLC patients who underwent surgery than AJCC stage. These tools may be helpful for clinicians to evaluate prognostic indicators of patients undergoing operation.

## 1. Introduction

NSCLC accounts for about 85% of all lung cancer, which remains the leading cause of cancer-related death in the world [1, 2]. In recent years, with the wide application of high-resolution spiral computed tomography (CT) screening technology, the detection rate of early lung cancer has increased significantly [3]. Surgery treatment is the first choice for patients diagnosed with early NSCLC, including stage I, stage II, and partial stage III cases. [4] The current treatment options for NSCLC mainly depend on the eighth edition of the American Joint Committee on Cancer TNM staging. However, patients' survival rate varies greatly at the same stage [5–7]. The 5-year survival rates range from 60% of stage I to about 30% of stage IIIA [8,9]. And patients with the same stage showed different rates of survival. It is of great significance in guiding clinical treatment to find independent prognostic factors. Previous studies [5–7] have reported that some factors may significantly promote the survival prediction of patients, such as age, race, sex, stage, and histology.

Nomogram is a convenient tool to predict and quantify risk for patients' prognosis by incorporating and validating some relevant factors. In some other types of tumors, nomograms that calculate numerical probability of clinical events, such as cancer-specific survival (CSS) and OS, have shown more precise prediction than the traditional TNM staging systems. At present, AJCC TNM staging is the main criterion to guide the treatment and prognosis of NSCLC patients. However, the staging could not be good to predict the survival for these patients. Other variables including age, sex, and histology may be significant independent prognostic factors for NSCLC patients. Therefore, the combination of AJCC staging and these variables may be better to predict the outcomes and it would be better in clinical guidance.

Therefore, in the present study, we built and validated the nomogram combined with several clinical variables to predict prognosis for patients with NSCLC who underwent surgery.

## 2. Materials and Methods

### 2.1. Data Source

The SEER Program (http://www.seer.cancer.gov) consists of 9 Regs Research Data in the United States [10]. Information for patients with stages I–III NSCLC between 2010 and 2015 was extracted from the SEER database. According to the AJCC criteria, we selected a total of 19617 patients diagnosed with NSCLC using the SEER^*∗*^Stat 8.3.5 software. The inclusion criteria for recruiting patients were as follows: NSCLC patients, only one malignant primary lesion, available clinical information, and active follow-up. The exclusion criteria were patients with benign tumor. In addition, patients containing any missing information on extracted data were all excluded.

### 2.2. Ethics Statement

Our study was constructed in accordance with the Helsinki Declaration. This study was also approved by the ethics committee of the Shandong Cancer Hospital. This study did not involve any personal information, and therefore, informed patient consent was not required.

### 2.3. Statistical Analysis

These eligible patients were randomly divided into the training cohort (70%, *n* = 13732) and the validation cohort (30%, *n* = 5885) to establish and validate the nomogram. The OS was defined as the time from diagnosis to death due to any reason. The data in training cohort were used to develop the prediction model and construct nomogram and risk classification system. Furthermore, the data of the validation cohort were used to make a validation.

Univariate and multivariate analyses were used to determine independent prognostic variables. And then, based on these variables contained in the final model, we built the nomogram and the risk classification system. The C-index was used to determine discrimination ability of the nomogram, and each parameter and ROC curves were used to evaluate the clinical utility of the nomogram. The calibration for 1-, 3-, and 5-year OS was evaluated using a calibration curve by comparing the predicted survival and the observed survival. Furthermore, based on the total score of each patient in the validation cohort, the risk classification system was established and all patients were divided into low-risk and high-risk prognosis groups. The OS was estimated using the Kaplan–Meier method and compared by the log-rank test.

All statistical analyses were made using R software version 3.4.3 (R Foundation) and Statistical Product Service Solutions (SPSS) 22.0 software package. All statistical *P* values were 2-sided, and *P* < 0.05 was considered statistically significant.

## 3. Results

### 3.1. Patients Characteristics

A total of 19617 patients initially diagnosed with NSCLC from the SEER database were included for analysis. All enrolled patients were randomly divided into the training cohort (13732, 70%) and the validation cohort (5885, 30%). According to age, all patients were divided into four groups including <60 years old (*n* = 4203, 21.4%), 60–69 years old (*n* = 7054, 36.0%), 70–79 years old (*n* = 6588, 33.6%), and >80 years old (*n* = 1772, 33.6%). In the total cohort, training cohort, and validation cohort, the proportion of patients aged 60–69 (36.0%, 36.1% and 35.6, respectively) was the largest. The majority of cases were white (*n* = 16312, 83.2%). Male and female patients accounted for the same proportion (50% vs. 50%).

According to the AJCC stage, patients of stage T1 were the largest in the total cohort, training cohort, and validation cohort (58.8%, 58.6%, and 59.4 respectively), followed by the T2 stage (23.3%, 23.5%, and 22.9%, respectively). And patients with stage T3 was the least in the total cohort, training cohort, and validation cohort (17.9%, 17.9%, and 17.7%, respectively). 12278 (62.6%) patients had adenocarcinoma and 7336 (37.4%) had squamous. 5.6% of patients underwent complete surgical resection, and 94.4% of patients underwent partial surgical resection. Of these patients, only 24.5% patients had positive lymph nodes. Baseline clinicopathological characteristics of all patients in the training cohort and the validation cohort are shown in Table 1.

### 3.2. Independent Prognostic Factors in Predicting OS

Univariate and multivariate Cox proportional hazards regression models were used to assess each factor's ability in predicting OS. In univariate analysis, we found that age (*P* < 0.001), race (*P* < 0.001), sex (*P*=0.03), stage (*P* < 0.001), histology (*P* < 0.001), surgery (*P* < 0.001), and positive lymph nodes (*P* < 0.001) were associated with OS in patients with stages I–III NSCLC. Among them, stage (C-index = 0.615), positive lymph nodes (C-index = 0.574), histology (C-index = 0.566), age (C-index = 0.563), and sex (C-index = 0.562) had superior discrimination power in predicting OS compared with other variables. Multivariate analysis further analyzed the factors of a *P* < 0.05 in univariate analysis. In the multivariate analysis, we found that age (*P* < 0.001), other races (*P* < 0.001), sex (*P* < 0.001), stage (*P* < 0.001), histology (*P* < 0.001), surgery (*P* < 0.001), and positive lymph nodes (*P* < 0.001) were independent prognostic factors and were incorporated into the predictive model. Univariate and multivariate analyses of each factor's ability in predicting OS are shown in Table 2.

### 3.3. Building and Validating the Predictive Nomogram

**Figure 1:**
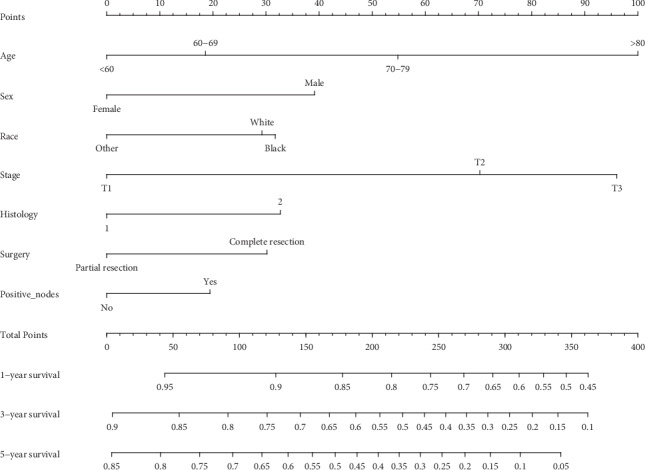
A nomogram for prediction of 1-, 3-, and 5-year OS rates of stages I–III NSCLC patients after surgery.

We built a novel nomogram that included the significant and independent prognostic factors (Figure 1). Each factor had a score on the point scale. We can draw a straight line to determine the estimated probability of prognosis at each time point by adding up the total score and locating it on the total point scale. And then, the validation cohort was used to verify the novel nomogram. In the validation cohort, we compared the OS rate predicted by the nomogram with observed 1-, 3-, and 5-year OS rates.

**Figure 2:**
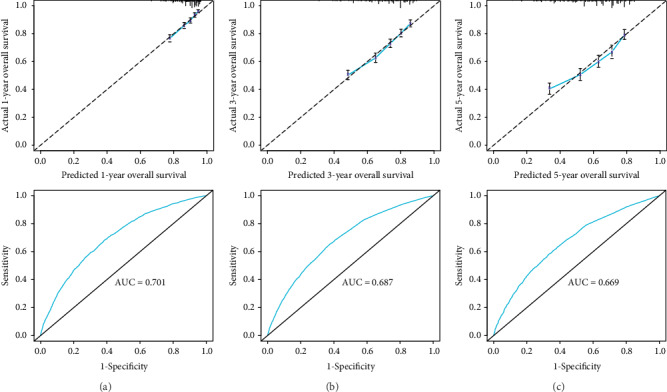
Calibration curves of the nomogram predicting 1-year, 3-year, and 5-year OS rates of stages I–III NSCLC patients after surgery. On the calibration plot, the *x*-axis is nomogram-predicted probability of over survival. The *y*-axis is the actual over survival.

In a well-calibrated model, the prediction will fall on a 45-degree diagonal line. From Figure 2, the calibration curves revealed good consistency between the actual observation and the nomogram prediction for 1-, 3-, and 5-year survival rates.  Figure 2(a) shows good consistency between the actual 1-year overall survival and predicted 1-year overall survival. And the ROC curve revealed that the area under the curve (AUC) is 0.701.  Figure 2(b) shows good consistency between the actual 3-year overall survival and predicted 3-year overall survival. And the ROC curve revealed that the AUC is 0.687.  Figure 2(c) shows good consistency between the actual 5-year overall survival and predicted 5-year overall survival. And the ROC curve revealed that the AUC is 0.669.

**Figure 3:**
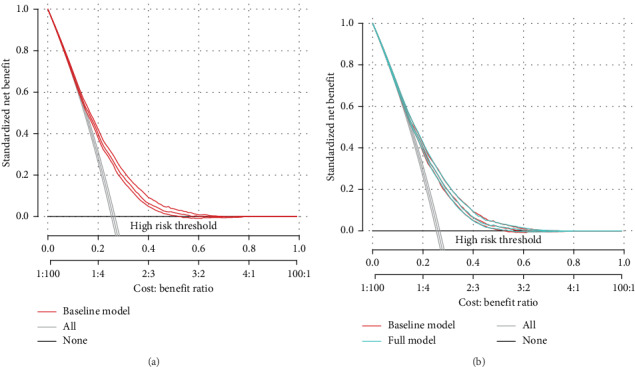
Decision curves of the nomogram predicting OS. The *x*-axis represents the threshold probabilities, and the *y*-axis measures the net benefit calculated by adding the true positives and subtracting the false positives.

In addition, decision curves exhibited great positive net benefits in the predictive model among almost all of the threshold probabilities at different time points, indicating the favorable potential clinical effect of the predictive model (Figures 3(a) and 3(b)).

### 3.4. Risk Classification System

**Figure 4:**
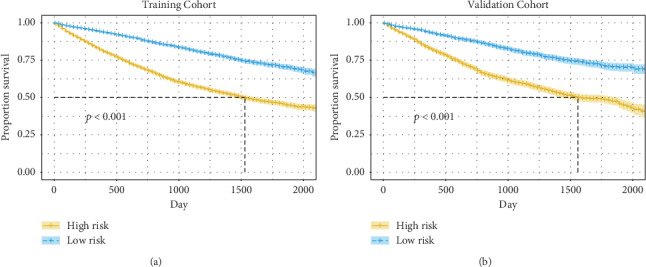
Kaplan–Meier curves of OS for patients in the low- and high-risk groups. (a) Kaplan–Meier curves of OS for patients in the low- and high-risk groups in the training cohort. (b) Kaplan–Meier curves of OS for patients in the low- and high-risk groups in the validation cohort.

According to the total scores of every patient, we also developed a risk classification system in the training cohort generated by nomogram. All patients in the training cohort and validation cohort were divided into the high-risk and low-risk groups. The Kaplan–Meier curve was used to draw the OS curves for the high-risk group and low-risk group in the training cohort and validation cohort. In the training cohort, the Kaplan–Meier curves revealed that patients' survival in the low-risk group was better than that in the high-risk group (*P* < 0.001) (Figure 4(a)). Then, we validated it in the validation cohort. Similarly, patients in the low-risk group had better survival than those in the high-risk group (*P* < 0.001) (Figure 4(b)).

## 4. Discussion

In this study, we established and developed a nomogram and a risk classification to predict the OS of patients with stages I–III NSCLC after surgery using the data originated from SEER database. A total of 19167 patients were included, and seven significant prognosis factors including age, race, sex, stage, histology, surgery, and positive nodes were identified. And these predictive factors could be easily obtained from clinical practice. Then, we established the validation of model and used different statistical methods to demonstrate its great performance.

Over time, the prospects for lung cancer patients and treatment have changed. Lung lobectomy is often considered the best treatment option for stages I, II, and partial III NSCLC patients [7,8,11]. Recurrence and metastasis have become important factors affecting the 5-year survival rate of patients with lung cancer after operation. So, it is very important to predict factors of survival after surgery in NSCLC patients. Furthermore, NSCLC has significant heterogeneity in individual survival, and it is inaccurate to use the TNM staging system to predict survival. Although several prognostic models have been reported previously [6,12], a relevant nomogram was rarely developed to predict prognostic variables for patients NSCLC after surgery.

Some research studies [13–18] reported that a nomogram could predict the prognosis of NSCLC patients. However, most studies focused on patients with early or advanced NSCLC. Nonetheless, both research studies had a small sample size which may inhibit their generalization.

Liang et al. [19] showed that the C-index for the established nomogram to predict OS was 0.71 in the primary cohort and 0.67 in the IASLC cohort. Sun et al. [13] showed that the C-index of the nomogram was 0.638 which exhibited a sufficient level of discrimination. However, in our study, the C-index of the nomogram is higher than that of other previous models. In addition to a nomogram, we also developed a risk classification system and the risk classification divided the whole NSCLC patients into two distinct prognostic groups which could supplement the nomogram in our study.

In univariable and subsequent multivariable analysis, we identified age, race, sex, stage, histology, surgery types, and positive lymph nodes as independent prognostic factors. These findings are consistent with previous reports on risk factors for non-small-cell lung cancer [7,8,20]. It is necessary to validate the nomogram and avoid excessive fitting of the model and determine the extensibility [11]. Notably, according to our nomogram, stage is the most powerful predictor of OS, and C-index (C-index = 0.615) was the highest among all predictors. One of the possible reasons is that TNM staging is the current important tool to make decision about the stage-specific therapeutic strategy and assess the prognostic survival [21]. However, in the present study, we did not divide these stages into specific T and N category, which were reported as the significant and independent factors in other research studies. We need future studies to assess each factor of stage which may impact on survival for patients with resected NSCLC.

In addition, positive lymph node was another important predictor for OS and the C-index was 0.574. Several research studies [22,23] reported the relationship between positive lymph nodes and survival. The reason may be that with more positive lymph nodes being cleared out, potential metastatic lymph nodes will be removed. For patients with resected NSCLC, the number of positive lymph nodes was also demonstrated as an important prognostic factor [24,25]. And in many other cancers, positive lymph node is an important factor affecting survival [26–28]. Moreover, complete sampling of lymph nodes results in precise staging and, therefore, appropriate adjuvant treatments for patients.

In this study, we defined 1-, 3-, and 5-year survival rates as our endpoints. Calibration curves showed good agreement between nomogram prediction and actual observation. The nomogram performed well by AUC at every measured time point, which revealed that the nomogram had good performance to predict 1-, 3-, and 5-year OS rates for patients with resected NSCLC. Kaplan–Meier curves showed that OS in the different groups was accurately differentiated by the risk classification system in the training cohort and validation cohort, both of *P* < 0.05.

Although surgery is the first choice treatment for patients with stages I, II, and partial III NSCLC [29, 30], postoperative adjuvant treatment could decrease the risk of disease recurrence and improve outcome [30–32]. It should be noted that postoperative adjuvant therapies including chemotherapy, radiotherapy, target treatment, and any other adjuvant therapies were not selected as candidate factors because they were only recommended for a proportion of patients with potentially high risk of locoregional recurrence.

In addition, patients with N2 disease were a heterogeneous group [33]. Operation may have some limitations for these patients, and the treatment should be individualized [34]. Mao et al. [35] showed that the C-index of the nomogram was 0.673 in the training cohort and 0.664 in the validation cohort. In our study, we did not specify the proportion of these patients with N2 disease who were treated with surgery from SEER database. The future studies are necessary to validate this result.

However, there are several limitations in our study. First, this was a retrospective study from the SEER database which could not represent the global population. Second, some other factors affecting survival, including smoking history, tumor location, and resection type, were not included in the present study. These data also may have an impact on clinical prognosis. Third, due to the limitations of the SEER database, the details of specific adjuvant therapy, such as chemotherapy and radiochemotherapy which may have some effect on survival for these patients, could not be obtained. Finally, although we use a large cohort to establish the nomogram and risk classification and validated in validation cohort, further validation of the predictive model is still essential.

## 5. Conclusion

We established a nomogram and a corresponding risk classification system predicting survival for NSCLC patients who underwent surgery. The results proved that the model had better performance to predict survival for NSCLC patients who underwent surgery than AJCC stage. Although future validation is necessary, these tools may be helpful for clinicians to evaluate prognostic indicators of patients undergoing operation.

## Figures and Tables

**Table 1:** Baseline clinicopathological characteristics of all patients and those in the training and validation cohorts.

Variables	All cohort (*n* = 19617)	Training cohort (*n* = 13732)	Validation cohort (*n* = 5885)	*P*
*Age*				0.026
<60	4203(21.4)	4958 (36.1)	1302 (22.1)	
60–69	7054(36.0)		2096 (35.6)	
70–79	6588(33.6)	4619 (33.6)	1969 (33.5)	
>80	1772(9.0)	1254 (9.1)	518 (8.8)	

*Race*				0.019
White	16312(83.2)	11445 (83.3)	4867 (82.7)	
Black	1814(9.2)	1262 (9.2)	552 (9.4)	
Others	1491(7.6)	1025 (7.5)	466 (7.9)	

*Sex*				0.013
Male	9807(50.0)	6839 (49.8)	2968 (50.4)	
Female	9810(50.0)	6893 (50.2)	2917 (49.6)	

*Stage*				0.017
I	11543(58.8)	8047 (58.6)	3496 (59.4)	
II	4572(23.3)	3226 (23.5)	1346 (22.9)	
III	3502(17.9)	2459 (17.9)	1043 (17.7)	

*Histology*				0.009
Adenocarcinoma	12278(62.6)	8579 (62.5)	3702 (62.9)	
Squamous	7336(37.4)	5153 (37.5)	2183 (37.1)	

*Surgery*				0.014
Complete resection	1092(5.6)	778 (5.7)	314 (5.3)	
Partial resection	18525(94.4)	12954 (94.3)	5571 (94.7)	
*Positive nodes*				0.005
Yes	4812(24.5)	3360 (24.5)	1452 (24.7)	
No	14805(75.5)	10372 (75.5)	4433 (75.3)	

**Table 2:** Univariate and multivariate analyses of each factor's ability in predicting OS.

	Univariate analyses				Multivariate analyses		
Variable	HR	95% CI	*P*	C-index	HR	95% CI	*P*
*Age*				0.563			
<60	Reference				Reference		
60–69	1.110	1.010–1.220	0.038		1.174	1.065–1.294	0.001
70–79	1.430	1.300–1.570	<0.001		1.604	1.455–1.768	<0.001
>80	2.00	1.780–2.260	<0.001		2.367	2.095–2.674	<0.001

*Race*				0.516			
White	Reference				Reference		
Black	0.913	0.813–1.025	0.120		1.022	0.909–1.148	0.717
Others	0.748	0.649–0.863	<0.001		0.777	0.673–0.897	<0.001

*Sex*				0.562			
Male	Reference				Reference		
Female	0.649	0.607–0.694	0.030	<0.001	0.714	0.667–0.764	<0.001

*Stage*				0.615			
I	Reference				Reference		
II	2.100	1.940–2.270	<0.001		1.832	1.672–2.006	<0.001
III	2.610	2.410–2.830	<0.001		2.287	2.047–2.554	<0.001

*Histology*				0.566			
Adenocarcinoma	Reference				Reference		
Squamous	1.570	1.470–1.6770	<0.001		1.325	1.237–1.420	<0.001

*Surgery*				0.528			
Complete resection	Reference				Reference		
Partial resection	1.990	1.780–2.230	<0.001		1.297	1.150–1.462	<0.001

*Positive nodes*			<0.001	0.574			
Yes	Reference				Reference		
No	2.030	1.900–2.170			1.183	1.077–1.299	<0.001

## Data Availability

The datasets used and analyzed during the current study are available from the corresponding author upon reasonable request.

## Abbreviations

NSCLC: Non-small-cell lung cancer
SEER: Surveillance epidemiology and end results
OS: Overall survival
CT: Computed tomography
HR: Hazard ratio
CI: Confidence interval
AJCC: American Joint Committee on Cancer
C-index: Concordance index
ROC: Receiver operating characteristic
AUC: Area under the curve.
